# Sarcoidosis presenting as fulminant bilateral multifocal choroiditis

**DOI:** 10.1186/s12348-026-00604-y

**Published:** 2026-06-06

**Authors:** Jason Tan, Ben Clark, Jordan Nizamovski, David Cordeiro Sousa

**Affiliations:** 1 Ophthalmology, University Hospital Geelong, Geelong, VIC Australia; 2 Department of Anatomical Pathology, Australian Clinical Labs, Geelong, VIC Australia; 3 Ophthalmology, Department of Surgery, University of Melbourne, Melbourne, VIC Australia; 4 Centre for Eye Research Australia, Royal Victorian Eye and Ear Hospital, Melbourne, VIC Australia

**Keywords:** Ocular sarcoidosis, Multifocal choroiditis, Inguinal lymph node biopsy, Extrapulmonary sarcoidosis, Uveitis

## Abstract

**Purpose:**

To report a case of fulminant bilateral multifocal choroiditis as the presenting manifestation of sarcoidosis despite normal serum angiotensin-converting enzyme (ACE) and unremarkable thoracic imaging.

**Case presentation:**

An immunocompetent 32-year-old man was admitted with a systemic inflammatory illness and peripheral lymphadenopathy. Given the acute systemic symptoms and recent international travel to Southeast Asia, an infectious cause was initially suspected. Two days after admission, he developed rapidly progressive bilateral central scotomas, floaters, and photophobia. Examination demonstrated bilateral granulomatous keratic precipitates, anterior chamber and vitreous inflammation, and multifocal yellowish deep retinal lesions with indistinct margins involving the posterior pole and midperiphery. Fundus autofluorescence demonstrated hypoautofluorescent lesions with surrounding hyperautofluorescence, and optical coherence tomography (OCT) showed retinal thickening with outer retinal disorganization, subretinal and intraretinal fluid, retinal pigment epithelium (RPE) hyperreflectivity and elevation, and choroidal thickening. Infectious investigations, neuroimaging, serum ACE, and thoracic imaging were unrevealing. Although vision deteriorated despite intensive topical therapy, it improved rapidly after high-dose systemic corticosteroids. Excisional biopsy of a palpable inguinal lymph node showed well-formed non-necrotizing granulomas with negative fungal and mycobacterial stains, findings considered in keeping with sarcoidosis. Methotrexate was commenced as steroid-sparing therapy. Quiescence was achieved within three weeks, and visual acuity recovered to 20/20 bilaterally by two months.

**Conclusions:**

Sarcoidosis may present as fulminant bilateral multifocal choroiditis even when serum ACE and thoracic imaging are normal. Careful general examination for peripheral lymphadenopathy, including inguinal nodes, may help identify an accessible extrathoracic biopsy target, facilitating timely tissue confirmation and early immunosuppression.

## Background

Sarcoidosis is a multisystem inflammatory disease characterized by noncaseating granulomas and can involve virtually any organ system [1]. Ocular involvement is common and may be the presenting manifestation [2–5]. Posterior segment phenotypes include retinal vasculitis, choroidal or optic nerve granuloma, and multifocal choroiditis, with the latter being reported as the most frequent posterior presentation of sarcoid-associated uveitis [2, 6, 7]. Since infectious posterior uveitis can mimic sarcoidosis and may worsen with corticosteroids, establishing the correct diagnosis is critical. The revised International Workshop on Ocular Sarcoidosis (IWOS) criteria emphasize tissue confirmation where possible, alongside a compatible clinical phenotype and exclusion of alternative causes [8]. However, reliance on pulmonary findings may delay diagnosis as sarcoidosis can present without intrathoracic lymphadenopathy or pulmonary parenchymal disease (radiographic stage 0), and a normal serum ACE cannot reliably exclude disease. Peripheral lymphadenopathy occurs in more than 20% of patients and can involve cervical, axillary, epitrochlear, and inguinal nodes [1]. In such settings, peripheral lymph node biopsy may provide diagnostic confirmation, including in patients without radiographic thoracic involvement [9]. Here, we report a case of fulminant bilateral multifocal choroiditis in which excisional inguinal lymph node biopsy enabled tissue confirmation and timely immunosuppression despite normal ACE and unremarkable thoracic imaging.

## Case presentation

A previously well, immunocompetent 32-year-old man developed a systemic inflammatory illness of unclear etiology. He presented with over a week of fluctuating headache with upper neck pain and mild blurred vision. Over the preceding days, he developed generalized tender lymphadenopathy involving the cervical, axillary, and inguinal regions, together with night sweats and chills, followed by pleuritic left anterior chest pain that was worse when lying flat and with deep inspiration, without preceding respiratory symptoms. He had a history of recent international travel to Southeast Asia but had returned well and reported no sick contacts. Given concern for an infectious cause, he was admitted, managed in airborne precautions, and commenced on empiric intravenous gentamicin and flucloxacillin while further investigations were undertaken. Electrocardiogram showed multi-territory ST-segment elevation with negative troponin and he was also treated for presumed pericarditis, with chest pain improving on colchicine and non-steroidal anti-inflammatory therapy.

Two days into his admission, he developed new visual disturbance, bilateral floaters and photophobia with ongoing retro-orbital headaches. This rapidly progressed to central scotomas and he was referred for urgent ophthalmic assessment. His best corrected vision was 20/100 in the right eye and 20/125 in the left. Intraocular pressure was 10 mmHg bilaterally. Pupils were equal with no relative afferent pupillary defect. Anterior segment examination demonstrated granulomatous keratic precipitates with 2 + anterior chamber cells in both eyes. Dilated fundus examination showed 2 + vitreous cells bilaterally and numerous yellowish, deep retinal lesions with fuzzy margins scattered throughout the posterior pole and midperiphery of both eyes. Topical prednisolone acetate 1% hourly and cycloplegia were commenced.

Systemic inflammatory markers were elevated (white blood count 9.3 × 10^9/L, erythrocyte sedimentation rate 40 mm/h, C‑reactive protein 148.1 mg/L). Lumbar puncture revealed clear cerebrospinal fluid with mild pleocytosis and a negative multiplex polymerase chain reaction panel for common bacterial and viral pathogens. Magnetic resonance imaging (MRI) of the brain and orbits was unremarkable. Infectious investigations relevant to posterior uveitis were negative, including blood cultures, human immunodeficiency virus, syphilis serology, QuantiFERON-TB, and toxoplasma serology. Serum ACE was within the reference range (28 U/L; reference 10–70). Computed Tomography (CT) of the chest, abdomen, and pelvis demonstrated no radiographically enlarged lymph nodes and no radiographic features of pulmonary sarcoidosis.

**Fig. 1:**
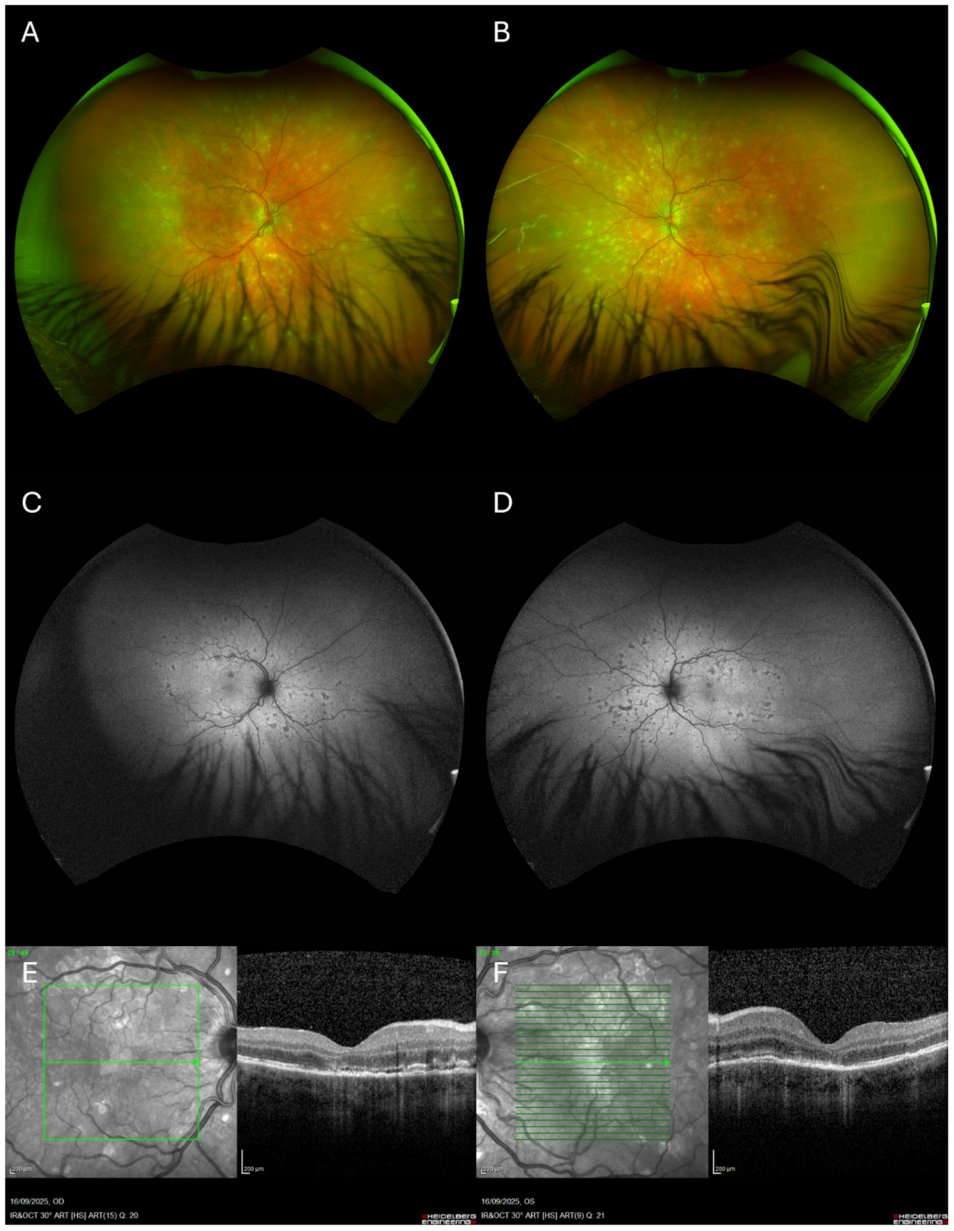
Baseline multimodal retinal imaging demonstrating active bilateral multifocal choroiditis. (**A, B**) On wide-field color fundus photography (right eye, **A**; left eye, **B**), bilateral yellowish deep retinal lesions with fuzzy margins are seen in the posterior pole and midperiphery. (**C, D**) On wide-field fundus autofluorescence (right eye, **C**; left eye, **D**), the yellowish lesions correspond to areas of hypoautofluorescence with surrounding hyperautofluorescence. (**E, F**) On macular spectral-domain OCT (right eye, **E**; left eye, **F**), there is retinal thickening, disorganization and loss of the outer retinal layers, subretinal and intraretinal fluid and hyperreflectivity of the RPE. RPE elevation suggests underlying choroidal granulomas. Choroidal thickening is noted

Despite intensive topical therapy, intraocular inflammation progressed over the subsequent three days, with visual acuity deteriorating to 20/250 in the right eye and 20/300 in the left eye. On wide-field fundus autofluorescence, the yellowish lesions corresponded to areas of hypoautofluorescence with surrounding hyperautofluorescence. Macular OCT demonstrated choroidal and retinal thickening, disorganization and loss of the outer retinal layers, subretinal and intraretinal fluid and hyperreflectivity of the RPE, consistent with active multifocal choroiditis (Fig. 1).

High-dose oral prednisolone (1 mg/kg/day) was commenced empirically due to the fulminant disease with rapidly progressive vision loss and the absence of evidence for an infectious etiology. In parallel, given the persistent palpable groin lymphadenopathy, targeted ultrasound identified an inguinal lymph node with preserved morphology but increased vascularity. Excisional inguinal lymph node biopsy was performed, with specimens submitted for histopathology, flow cytometry, and mycobacterial studies.

**Fig. 2:**
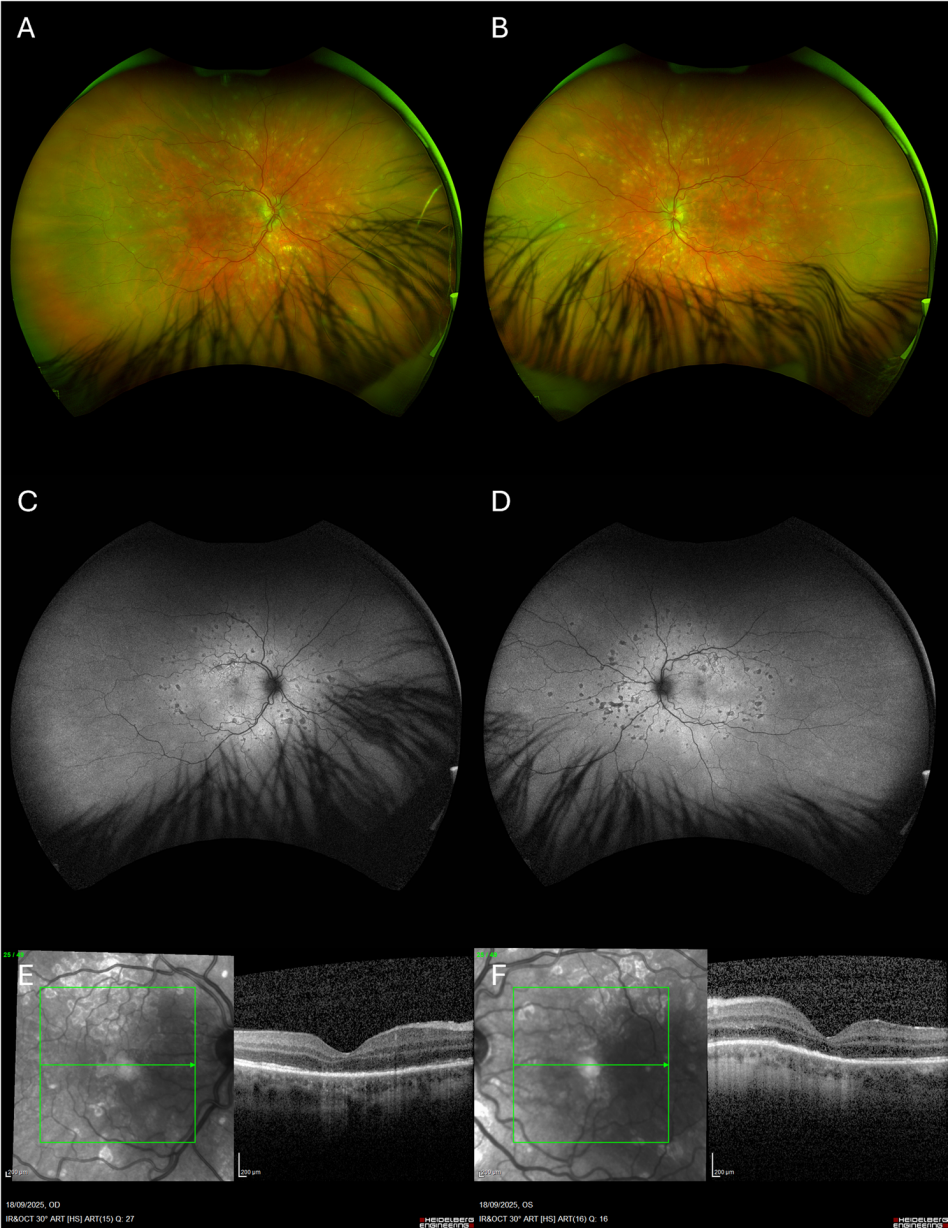
Early follow-up (48 h) multimodal imaging demonstrating rapid anatomic improvement after systemic corticosteroid initiation. (**A, B**) On wide-field color fundus photography (right eye, **A**; left eye, **B**), bilateral multifocal lesions are more well-defined and less clinically active following treatment. (**C, D**) On wide-field fundus autofluorescence (right eye, **C**; left eye, **D**), the inactive lesions become predominantly hypoautofluorescent with well-defined margins and marked reduction of the surrounding hyperautofluorescence. (**E, F**) On macular spectral-domain OCT (right eye, **E**; left eye, **F**), there is marked reduction in subretinal and intraretinal fluid, with improving outer retinal architecture and reduced RPE and choroidal abnormalities

A favorable anatomic and functional response was observed 48 h after initiating high-dose corticosteroids. The multifocal lesions became more well defined and less clinically active. Fundus autofluorescence showed predominantly hypoautofluorescent lesions with marked reduction of the surrounding hyperautofluorescence. Macular OCT demonstrated marked reduction in subretinal and intraretinal fluid, with improving outer retinal architecture and reduced RPE and choroidal abnormalities (Fig. 2).

**Fig. 3:**
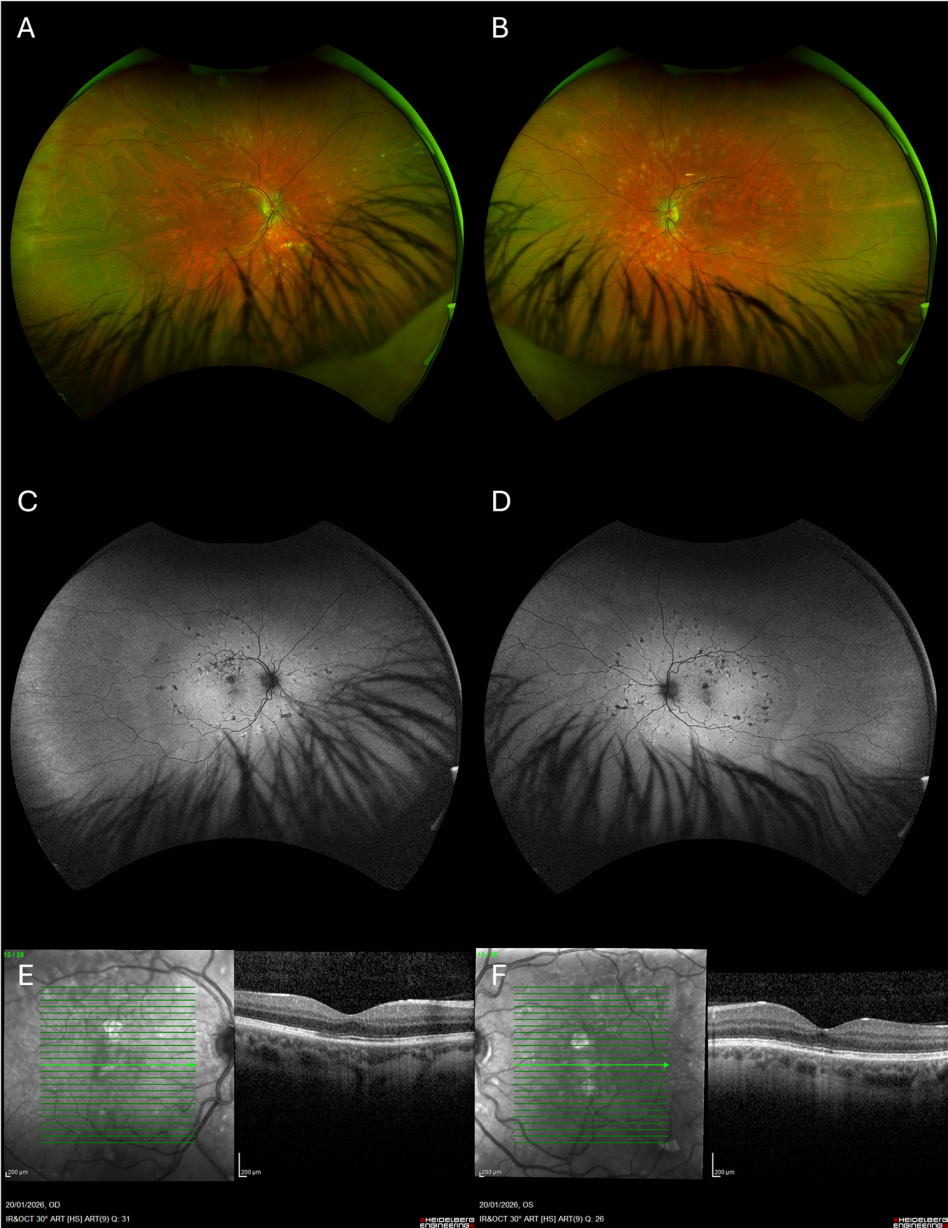
Excisional biopsy of a palpable right inguinal lymph node demonstrating non-necrotizing granulomatous inflammation. (**A**) Low-power histopathology image shows scattered, well-formed epithelioid granulomas within lymph node parenchyma. (**B**) Higher-power image shows epithelioid macrophages and occasional multinucleated giant cells. No necrosis or suppurative inflammation is seen. PAS and acid-fast bacilli stains were negative for fungal or mycobacterial organisms

Histopathology of the excised inguinal lymph node showed a fragmented lymph node containing scattered well-formed non-necrotizing epithelioid granulomas in a background of small lymphocytes, without necrosis or suppurative inflammation. Occasional multinucleated giant cells were present. No large atypical lymphoid cells or metastatic malignancy were identified. Periodic Acid-Schiff (PAS) and acid-fast bacilli stains showed no fungal or mycobacterial organisms, and immunohistochemistry (CD3, CD5, CD20) demonstrated a mixed population of B and T cells. Although these findings were not specific for sarcoidosis in isolation, in the clinical context they were considered supportive of sarcoidosis (Fig. 3). Methotrexate with folate supplementation was commenced as steroid-sparing therapy.

**Fig. 4:**
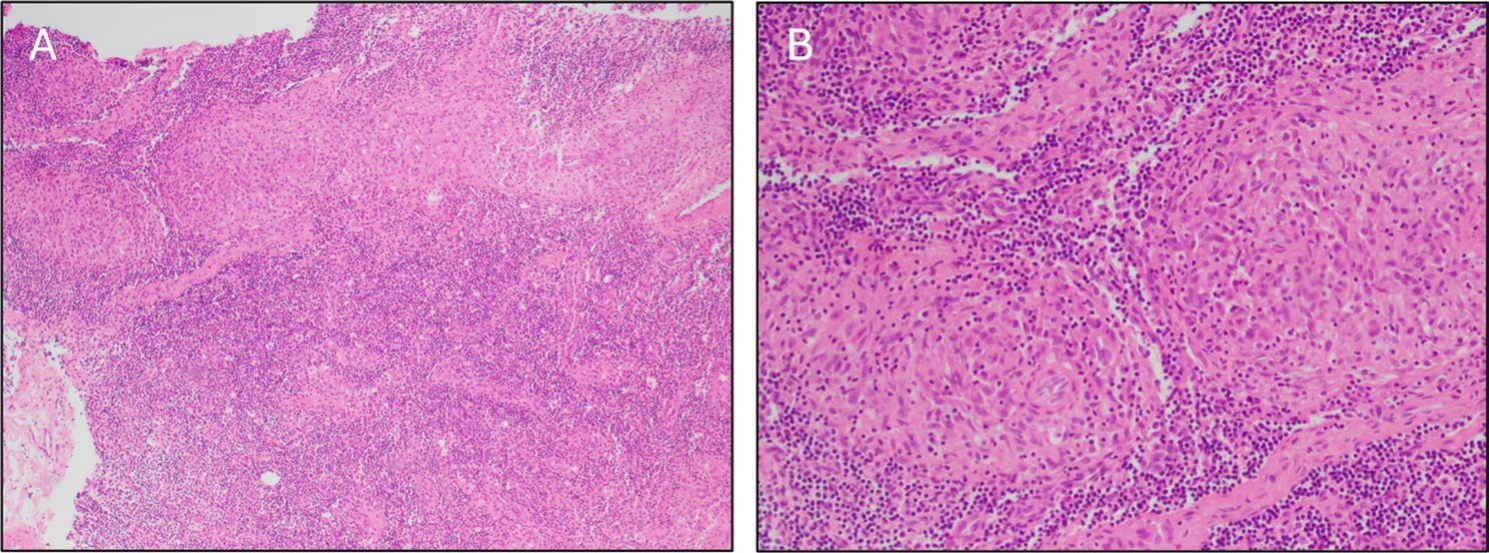
Late follow-up (two months) multimodal imaging demonstrating quiescent bilateral multifocal choroiditis with inactive chorioretinal scarring. (**A, B**) On wide-field color fundus photography (right eye, **A**; left eye, **B**), bilateral multifocal lesions are well-defined, inactive scars in the posterior pole and midperiphery. (**C, D**) On wide-field fundus autofluorescence (right eye, C; left eye, D), the inactive lesions become predominantly hypoautofluorescent with well-defined margins and disappearance of surrounding hyperautofluorescence. (**E, F**) On macular spectral-domain OCT (right eye, **E**; left eye, **F**), the subretinal and intraretinal fluid, with improving outer retinal architecture and reduced RPE and choroidal abnormalities have resolved with treatment

Quiescence was achieved within three weeks of initiating systemic corticosteroids, allowing gradual tapering. Visual acuity recovered to 20/20 bilaterally by two months, with evolution of the remaining active lesions into more well-defined, inactive scars. Fundus autofluorescence showed predominantly hypoautofluorescent lesions with well-defined margins and disappearance of the surrounding hyperautofluorescence. OCT confirmed resolution of subretinal and intraretinal fluid with improvement in outer retinal architecture and residual outer retinal and RPE irregularity corresponding to healed inflammatory lesions (Fig. 4).

## Discussion

This case highlights several practical points for clinicians managing severe multifocal choroiditis.

Ocular involvement is the presenting manifestation in approximately 20 to 30% of patients with sarcoidosis, and ophthalmic findings may therefore precede systemic confirmation of disease [3–5]. This is relevant to this case, as fulminant bilateral multifocal choroiditis was the initial manifestation leading to eventual histopathologic confirmation. Sarcoidosis should therefore remain an important differential diagnosis in bilateral multifocal choroiditis, particularly when accompanied by granulomatous anterior segment inflammation and vitritis. This phenotype has a recognized association with sarcoidosis, with a substantial proportion of cases classified as proven or presumed sarcoidosis [2, 6, 7]. Although many reported cohorts describe older, predominantly female populations, this presentation shows that fulminant disease with rapid visual decline can also occur in younger male patients [6, 7].

This case also underscores the diagnostic value of actively searching for an accessible extrathoracic tissue target when initial investigations are nondiagnostic. The IWOS criteria provide a structured approach, but definitive classification rests on histopathologic confirmation of noncaseating granulomas with exclusion of infectious mimics and other alternative causes [8]. Importantly, non-necrotizing granulomas are not specific for sarcoidosis and may also be seen with infection (including mycobacterial or fungal), autoimmune disease such as Crohn’s disease, and foreign body reaction. Accordingly, special stains, microbiological studies, and clinicopathological correlation remain essential. In our case, PAS and acid-fast bacilli stains were negative, no metastatic carcinoma or lymphoma was identified, and the lymph node showed a mixed B- and T-cell population on immunohistochemistry, supporting a reactive granulomatous process rather than malignancy. Sarcoidosis may also present without intrathoracic lymphadenopathy or pulmonary parenchymal disease (radiographic stage 0), and a normal serum ACE does not exclude the diagnosis [1]. In this context, a careful general examination for peripheral lymphadenopathy is essential, as superficial nodes may provide a low-morbidity route to tissue confirmation and avoid delays associated with pursuing thoracic targets. Inguinal lymphadenopathy may be overlooked unless specifically examined for; however, when present, inguinal nodes can provide diagnostic tissue with high utility, including in patients without radiographic thoracic involvement [9]. In our patient, recognition of persistent palpable groin lymphadenopathy, supported by ultrasound, enabled excisional biopsy that supported the diagnosis, excluded malignant and mycobacterial mimics and facilitated timely immunotherapy.

Early systemic therapy can be vision-saving in fulminant sarcoid choroiditis. Systemic corticosteroids with steroid-sparing immunomodulatory therapy (commonly methotrexate) are frequently used to facilitate corticosteroid taper while maintaining control of inflammation [1, 7]. The rapid structural response within 48 h and complete visual recovery by two months illustrate the potential for good outcomes when timely immunosuppression is instituted after infection has been reasonably excluded.

In summary, in patients with sight threatening bilateral multifocal choroiditis and systemic symptoms, normal thoracic imaging and a normal serum ACE do not exclude sarcoidosis. Careful general examination for peripheral lymphadenopathy may identify an accessible extrathoracic biopsy target, even in the absence of intrathoracic disease, and thereby facilitate timely diagnosis and earlier immunosuppression.

## Data Availability

No datasets were generated or analysed during the current study.
